# Safety and pharmacokinetics of motesanib in combination with gemcitabine and erlotinib for the treatment of solid tumors: a phase 1b study

**DOI:** 10.1186/1471-2407-11-313

**Published:** 2011-07-26

**Authors:** Dusan Kotasek, Niall Tebbutt, Jayesh Desai, Stephen Welch, Lillian L Siu, Sheryl McCoy, Yu-Nien Sun, Jessica Johnson, Adeboye H Adewoye, Timothy Price

**Affiliations:** 1 Adelaide Cancer Center, Level 1, Tennyson Centre, 520 South Road, Kurralta Park, SA 5037, Australia; 2 Medical Oncology Unit, Level 6, Harold Stokes Building, Austin Hospital, 145 Studley Road, Heidelberg, VIC 3084, Australia; 3 Department of Medical Oncology, The Royal Melbourne Hospital, Grattan Street, Parkville, VIC 3050, Australia; 4 The Princess Margaret Hospital, 610 University Avenue, Toronto, Ontario M5G 2M9, Canada; 5 Amgen Inc., One Amgen Center Drive, Thousand Oaks, CA, 91320-1799, USA; 6 Department of Medical Oncology, The Queen Elizabeth Hospital, 28 Woodville Road, Woodville South, SA 5011, Australia

## Abstract

**Background:**

This phase 1b study assessed the maximum tolerated dose (MTD), safety, and pharmacokinetics of motesanib (a small-molecule antagonist of VEGF receptors 1, 2, and 3; platelet-derived growth factor receptor; and Kit) administered once daily (QD) or twice daily (BID) in combination with erlotinib and gemcitabine in patients with solid tumors.

**Methods:**

Patients received weekly intravenous gemcitabine (1000 mg/m^2^) and erlotinib (100 mg QD) alone (control cohort) or in combination with motesanib (50 mg QD, 75 mg BID, 125 mg QD, or 100 mg QD; cohorts 1-4); or erlotinib (150 mg QD) in combination with motesanib (100 or 125 mg QD; cohorts 5 and 6).

**Results:**

Fifty-six patients were enrolled and received protocol-specified treatment. Dose-limiting toxicities occurred in 11 patients in cohorts 1 (n = 2), 2 (n = 4), 3 (n = 3), and 6 (n = 2). The MTD of motesanib in combination with gemcitabine and erlotinib was 100 mg QD. Motesanib 125 mg QD was tolerable only in combination with erlotinib alone. Frequently occurring motesanib-related adverse events included diarrhea (n = 19), nausea (n = 18), vomiting (n = 13), and fatigue (n = 12), which were mostly of worst grade < 3. The pharmacokinetics of motesanib was not markedly affected by coadministration of gemcitabine and erlotinib, or erlotinib alone. Erlotinib exposure, however, appeared lower after coadministration with gemcitabine and/or motesanib. Of 49 evaluable patients, 1 had a confirmed partial response and 26 had stable disease.

**Conclusions:**

Treatment with motesanib 100 mg QD plus erlotinib and gemcitabine was tolerable. Motesanib 125 mg QD was tolerable only in combination with erlotinib alone.

**Trial Registration:**

ClinicalTrials.gov NCT01235416

## Background

The development of targeted therapies has greatly improved treatment for many types of cancers [1]. Specifically, inhibitors of vascular endothelial growth factor (VEGF) signaling, including monoclonal antibodies targeting VEGF and small molecules targeting VEGF receptors (VEGFR), have demonstrated efficacy in the treatment of a variety of solid tumors [2-5]. Similarly, inhibitors of the epidermal growth factor receptor (EGFR) have shown clinical efficacy in the same setting [6-8].

In an effort to increase treatment benefits, combinations of targeted therapies are currently being explored. In phase 1 and 2 studies of advanced non—small cell lung cancer (NSCLC), treatment with the VEGF inhibitor bevacizumab plus erlotinib resulted in response rates of 17.5% to 20.0% [9-11]. In a phase 3 study of patients with advanced NSCLC in whom first-line therapy previously failed [12], treatment with this combination resulted in improved progression-free survival (3.4 vs 1.7 mo; hazard ratio [HR], 0.62; 95% confidence interval [CI], 0.52-0.75; *P *< 0.0001) and response rate (12.6% vs 6.2%; *P *= 0.006) compared with patients who received erlotinib alone. However, no effect on overall survival was observed (9.3 vs 9.2 mo; HR, 0.97; 95% CI, 0.80-1.18; *P *= 0.75). Erlotinib (100 mg/day) plus gemcitabine is indicated in the first-line treatment of locally advanced, unresectable or metastatic pancreatic cancer [13]. Potentially, the addition of a VEGF pathway inhibitor might improve outcomes beyond that achieved with erlotinib plus gemcitabine.

Motesanib is an orally administered small-molecule antagonist of VEGFR 1, 2, and 3; platelet-derived growth factor receptor; and Kit [14]. In preclinical A431 human epidermoid carcinoma, HT29 colorectal carcinoma, and Calu-6 NSCLC xenograft models, administration of motesanib in combination with the fully human anti-EGFR monoclonal antibody panitumumab resulted in greater antitumor activity than single-agent treatment [15]. In clinical studies conducted in patients with solid tumors, motesanib has demonstrated antitumor activity as monotherapy [16,17], in combination with cytotoxic chemotherapy [18,19], and, in lung and colorectal cancers, in combination with panitumumab (a fully human anti-EGFR antibody) and chemotherapy [19,20]. The present phase 1b study explored the feasibility of combination treatment strategies with motesanib, gemcitabine, and erlotinib in patients with solid tumors. The study objectives were to determine the target or maximum tolerated dose (MTD) and to characterize the safety and pharmacokinetics of motesanib administered once daily (QD) or twice daily (BID) in combination with erlotinib and gemcitabine in patients with solid tumors.

## Methods

### Patients

Eligible patients (aged > 18 years) had histologically or cytologically documented solid tumors, had an Eastern Cooperative Oncology Group performance status ≤ 2, and were candidates for treatment with erlotinib or with the combination of gemcitabine and erlotinib in the opinion of the investigator. Key exclusion criteria were: squamous cell NSCLC; hematologic malignancies; large central thoracic tumor lesions; direct bowel wall invasion (except for primary tumors of the bowel); untreated or symptomatic brain metastases; primary solid cancers with no known active disease present and no curative treatment administered for the last 3 years (except for curatively treated nonmelanoma skin cancer); history of bleeding or bleeding diathesis, or arterial or deep vein thrombosis; myocardial infarction within 1 year of study enrollment; uncontrolled hypertension (systolic blood pressure > 145 mmHg and diastolic blood pressure > 85 mmHg); inadequate cardiac, hepatic, renal or hematologic function; prior treatment with VEGF/VEGFR inhibitors, erlotinib, or gemcitabine; and systemic chemotherapy (within 21 days of study enrollment) or radiation therapy (within 14 days of study enrollment). All patients provided written informed consent, and ethical approval was obtained for all study procedures from each participating center's independent ethics committee or institutional review board. The study was conducted according to the Declaration of Helsinki.

### Study design

This was a phase 1b open-label dose-finding study of motesanib in combination with erlotinib and gemcitabine or with erlotinib alone in patients with advanced solid tumors, conducted at 4 study centers in Australia and Canada. The primary endpoint was the incidence of dose-limiting toxicities (DLTs; defined below); the secondary endpoint was determination of the pharmacokinetic profiles of motesanib and erlotinib. The safety endpoint was the incidence of adverse events. Tumor response rate, as assessed by Response Evaluation Criteria in Solid Tumors (RECIST) [21] in patients with measurable disease, was an exploratory endpoint.

**Figure 1:**
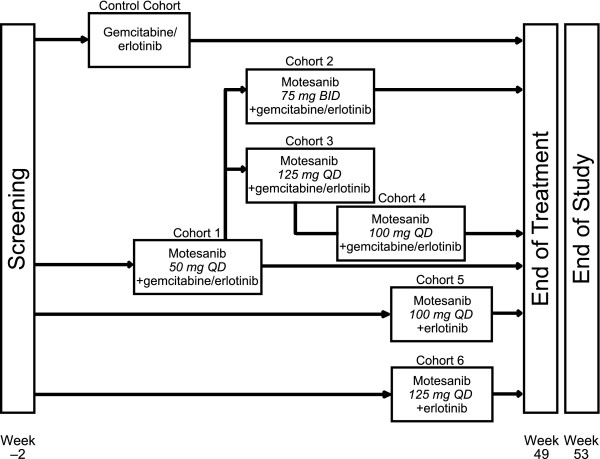
**Study schema**.

The study enrolled a control cohort (erlotinib 100 mg QD plus gemcitabine) and four motesanib dose escalation cohorts (cohorts 1-4), in which patients received motesanib plus erlotinib (100 mg QD) and gemcitabine. Once the motesanib MTD was determined, cohorts 5 and 6 were enrolled receiving erlotinib (150 mg QD) plus motesanib at the MTD (cohort 5) or at a higher dose (cohort 6) (Figure 1). Enrollment of a minimum of six evaluable patients per cohort was planned. If patients discontinued the study before week 5 for any reason other than a DLT, additional patients could be enrolled to meet this goal. The selected motesanib doses (50 mg QD, 100 mg QD, 125 mg QD, and 75 mg BID) were based on the tolerability profiles of motesanib administered as monotherapy [16] and in combination with gemcitabine [18] that were obtained in previous studies. The BID dosing regimen is expected to achieve higher predose motesanib concentrations in the plasma than QD dosing owing to the higher total dose administered and more frequent dosing.

### Treatment, dose escalation and maximum tolerated dose

Patients in the control cohort and cohorts 1 to 4 received erlotinib (100 mg QD throughout the study) and gemcitabine (1000 mg/m^2^) intravenously weekly for 7 weeks (cycle 1) or 3 weeks (cycles 2-11), followed by 1 week of rest. Treatment continued until the end of week 48 (11 cycles), or until disease progression, death, or unacceptable toxicity occurred. Motesanib administration (in cohorts 1-4) began on day 2 of week 2 of the first cycle and continued throughout the study. Patients experiencing continuous clinical benefit (stable disease or response) at the end of 48 weeks were eligible to continue motesanib monotherapy under a separate protocol.

Patients enrolled in cohort 1 received an initial dose of motesanib of 50 mg QD. If ≤ 2 patients experienced a DLT during the first 5 weeks of treatment, enrollment into cohort 2 (motesanib 75 mg BID) and cohort 3 (motesanib 125 mg QD) began simultaneously. If ≤ 2 patients in cohort 3 experienced a DLT in the first 5 weeks of treatment, the 125-mg QD dose would be considered the target once-daily dose. If ≥ 3 patients in cohort 3 had a DLT in that time frame, cohort 4 (motesanib 100 mg QD) was enrolled. The 100-mg QD dose would be considered the motesanib MTD if ≤ 2 patients in cohort 4 had a DLT in the first 5 weeks of treatment. After the MTD of motesanib was established, patients were enrolled in two additional cohorts receiving a higher dose of erlotinib (150 mg QD) plus motesanib at the MTD (100 mg QD; cohort 5) or at a higher dose (125 mg QD; cohort 6) without gemcitabine. Enrollment into cohort 6 started only after the lower motesanib dose administered in cohort 5 was determined to be safe in combination with erlotinib 150 mg QD.

Motesanib and erlotinib treatment was modified or withheld according to protocol-specified rules. Briefly, motesanib treatment was withheld for suspected or related grade 3 toxicity (other than hypertension) not adequately controlled with supportive care, or treatment-related grade 4 toxicity. In patients with symptomatic hypertension that required immediate or urgent management, motesanib treatment was withheld and antihypertensive medications were initiated or optimized. In patients with erlotinib-related toxicity, the erlotinib dose could be decreased in 50-mg decrements (no dose re-escalation was allowed).

Patients were permanently withdrawn from treatment if motesanib was withheld for > 3 continuous weeks, if more than two 25-mg dose reductions were required, or if symptomatic grade 4 venous thrombosis or grade 3 or 4 arterial thrombosis developed. Likewise, doses of erlotinib and gemcitabine could be modified based on protocol-specified rules.

### Adverse events and dose-limiting toxicities

All adverse events were graded by the investigator according to National Cancer Institute Common Terminology Criteria for Adverse Events (CTCAE) version 3.0. All adverse events were classified according to relatedness to treatment and seriousness. Events were considered related based on the investigator's assessment that the event may possibly have been caused by the treatment. Adverse events typically considered to be related to motesanib (or erlotinib) included events common to the pharmacologic class of VEGFR or multikinase (or EGFR) inhibitors and events that have been previously associated with motesanib (or erlotinib). A DLT was defined as treatment-related grade 3 fatigue for ≥ 7 days or grade 4 fatigue; grade 3 or 4 nausea/vomiting despite maximum supportive care; grade 3 neutropenia with fever > 38.5°C or grade 4 neutropenia; grade 4 thrombocytopenia for ≥ 7 days; grade 4 anemia; grade 4 hypertension; alanine aminotransferase or aspartate aminotransferase > 10 times the upper limit of normal; grade 3 rash for ≥ 7 days despite maximum supportive care or grade 4 rash; grade 3 diarrhea for ≥ 7 days despite maximum supportive care or grade 4 diarrhea; or any other treatment-related hematologic or nonhematologic grade 3 or 4 toxicity (except alopecia) occurring in the first 5 weeks of treatment.

### Pharmacokinetic analyses

Plasma samples for erlotinib pharmacokinetic analysis were collected predose and at 0.25, 0.5, 1, 2, 4, and 6 hours postdose on study day 8 (week 2) and predose on study day 9 (24 hours after day 8 dose) to ensure that steady state was reached. Plasma samples for both erlotinib and motesanib pharmacokinetic analysis were collected predose on study days 10 and 12; predose and at 0.25, 0.5, 1, 2, 4, 6, and 12 hours (BID cohort only) postdose on study day 15 (week 3); and predose on study day 16 (24 hours post day 15 dose).

Plasma concentrations of motesanib were analyzed using a validated liquid chromatography/tandem mass spectrometry (LC-MS/MS) method with a lower limit of quantitation (LLOQ) of 0.2 ng/mL (Cedra Corp., Austin, TX). Erlotinib concentrations were also assessed using a validated LC-MS/MS procedure (LLOQ, 50 ng/mL; Charles River Laboratories, Worcester, MA, and Shrewsbury, MA).

Pharmacokinetic parameter estimates were calculated according to standard noncompartmental methods using WinNonlin software (version 5.1.1; Pharsight Corporation, Mountain View, CA) and summarized by dose level. For both motesanib and erlotinib, maximum observed plasma concentration (C_max_) and the time to reach C_max _(t_max_) were taken directly from the plasma concentration-time data. The area under the concentration versus time curve (AUC) from time 0 to 24 hours (AUC_0—24_) after dosing was calculated using the linear-log trapezoidal rule. For the BID cohort, AUC_0—24 _values for motesanib were determined by 2 × AUC_0—12. _The terminal elimination rate constant (λ_Z_) was determined by linear regression of the natural logarithms of plasma concentrations versus time during the terminal phase; the corresponding t_1/2 _was calculated.

To assess the effect of motesanib administration on erlotinib exposure with or without gemcitabine, geometric least squares means (GLSM) and the ratio of GLSM between week 3 (with motesanib) and week 2 (erlotinib alone at steady state) were calculated for C_max _and AUC_0—24 _using the SAS PROC MIXED procedure (SAS for Windows, version 9.1, WIN_PRO platform; SAS Institute Inc., Cary, NC). The GLSM were calculated by first obtaining the least squares means for the log-transformed C_max _values (logC_max_) and AUC_0—24 _values (logAUC_0—24_) for weeks 3 and 2, and then converting these values back to the original scale. The ratio and 90% confidence interval (CI) of GLSM was calculated by first estimating the difference (and 90% CI) in the least squares means between weeks 3 and 2 for logC_max _and logAUC_0—24_, and then converting the numbers back to their original scale. The pharmacokinetic analysis included all patients who received motesanib and erlotinib with or without gemcitabine and who had evaluable plasma samples; the GLSM analysis included only patients with available pharmacokinetic parameter data for the protocol-specified treatment.

### Tumor response

Tumor assessments were performed using computed tomography or magnetic resonance imaging at baseline and at least every 12 weeks after the initial scan and included all sites of disease. Objective response was confirmed at least 4 weeks after the initial scan. Tumor assessments were performed by the investigator according to RECIST [21]. The analysis of response included all patients with measurable disease at baseline.

### Statistical analysis

The planned minimum sample size was 24 patients (6 evaluable patients in a minimum of 3 dose-escalation cohorts and a control cohort). Cohort enrollment could be expanded to ensure that at least 6 evaluable patients were enrolled or in the event of unresolved safety, pharmacokinetic, or other concerns. The overall sample size could be further increased if additional dose cohorts were considered justified based on the results from the dose-escalation portion of the study. For continuous endpoints, the mean, standard error or standard deviation (SD); median; 25th and 75th percentiles; and minimum and maximum were calculated. For discrete data, the frequency and percent distributions were calculated. No formal comparisons between cohorts were performed. The data were analyzed using SAS software version 8.2 (SAS Institute, Cary, NC). The statistical analysis was completed on March 20, 2009. The safety analysis set included all patients who received at least 1 dose of study therapy.

## Results

### Patients

From September 2006 to September 2007, 57 patients were enrolled in the study and 56 patients received study treatment, including eight patients in the control cohort. Demographic and baseline characteristics are summarized in Table 1. The main reasons for discontinuing treatment with motesanib, erlotinib, or gemcitabine were disease progression (n = 25, n = 37, n = 24; respectively), adverse events (n = 9, n = 7, n = 8), and withdrawal of consent (n = 4, n = 5, n = 5).

**Table 1:** Demographic and clinical characteristics of study patients

	Gemcitabine + Erlotinib (100 mg QD)	Motesanib + Gemcitabine + Erlotinib (100 mg QD)				Motesanib + Erlotinib (150 mg QD)	
			
Characteristic	Control (n = 8)	Motesanib 50 mg QD (n = 7)	Motesanib 100 mg QD (n = 8)	Motesanib 125 mg QD (n = 10)	Motesanib 75 mg BID (n = 9)	Motesanib 100 mg QD (n = 7)	Motesanib 125 mg QD (n = 7)
Sex, n							
Women	3	6	3	5	7	7	2
Men	5	1	5	5	2	0	5
Race, n							
White	7	7	6	10	6	6	7
Asian	0	0	2	0	3	1	0
Japanese	1	0	0	0	0	0	0
Median age, y (range)	62 (36-71)	66 (46-80)	50 (40-75)	59.5 (36-77)	53 (21-76)	50 (35-76)	55 (46-73)
ECOG performance status, n							
0	3	1	5	4	4	5	5
1	5	5	3	5	5	2	2
2	0	1	0	1	0	0	0
Disease stage, n							
I	0	0	0	1	0	0	0
II	0	0	0	1	0	0	1
III	0	0	1	0	1	0	0
IV	8	7	7	8	8	7	6
Tumor type, n							
Colon	3	0	1	1	1	1	0
Pancreatic	1	1	0	1	0	0	2
Breast	0	0	1	0	0	2	1
Melanoma	0	0	1	1	1	0	0
Non—small							
cell lung	0	1	1	0	1	0	0
Squamous cell							
head/neck	0	1	1	0	0	1	0
Medullary							
thyroid	0	0	1	1	0	0	1
Carcinoma							
unknown							
origin	1	0	0	1	0	0	0
Ovarian	0	1	0	0	1	0	0
Prostate	0	0	0	0	0	0	2
Stomach	0	1	0	0	1	0	0
Other^a^	3	2	2	5	4	3	1
Prior therapy, n^b^							
0	3	1	0	6	2	1	1
1 to 2	1	2	3	1	4	1	1
3 to 4	1	2	2	1	0	1	3
≥ 5	3	2	3	2	3	4	2
Prior chemotherapy, n							
0	3	2	2	8	2	1	1
1 to 2	1	3	3	1	4	2	3
3 to 4	1	1	2	1	1	1	1
≥ 5	3	1	1	0	2	3	2
Prior radiotherapy, n							
0	5	3	3	6	5	5	4
1 to 2	3	3	3	3	3	1	2
3 to 4	0	1	2	0	1	1	1
≥ 5	0	0	0	1	0	0	0

BID = twice daily; ECOG = Eastern Cooperative Oncology Group; QD = once daily

^a^Includes tumor types occurring in ≤ 2% of patients.

^b^All cancer therapies patients received before study enrollment.

At the time of data cutoff (November 2008), one patient continued to receive treatment. In cohorts receiving motesanib, erlotinib, and gemcitabine, the median treatment duration was 75 days (range, 17-653 days) for motesanib and 75.5 days (range, 1-346 days) for erlotinib; the median number of gemcitabine infusions was 10 (range, 3-54). In cohorts receiving motesanib and erlotinib only, the median treatment duration was 70.5 days (range, 19-244 days) for motesanib and 78.5 days (range, 27-252 days) for erlotinib. Median follow-up time was 18 weeks (range, 2-82 weeks).

### Dose escalation, dose-limiting toxicities, and maximum tolerated dose

Seven patients were enrolled in cohort 1 (motesanib 50 mg QD), two of whom experienced DLTs (one patient had grade 4 febrile neutropenia and one had grade 3 fatigue). Consequently, cohorts 2 (75 mg BID) and 3 (125 mg QD) were opened simultaneously and enrolled nine and 10 patients, respectively. In cohort 2, four patients experienced DLTs (including grade 3 nausea, tumor necrosis, and rash; and grade 4 neutropenia). In cohort 3, three patients had DLTs (including grade 3 nausea, vomiting, fatigue, cholecystitis, jaundice, subdural hematoma, and cognitive disorder); therefore, cohort 4 administering a lower dose of motesanib (100 mg QD) was opened and nine patients were enrolled (one patient did not receive treatment). No DLTs occurred and thus the MTD of motesanib in combination with gemcitabine and erlotinib was established as 100 mg QD. Subsequently, cohorts 5 and 6 were opened, enrolling seven patients each. In cohort 5, patients received erlotinib 150 mg QD plus motesanib at the MTD (100 mg QD); in cohort 6, patients received erlotinib 150 mg QD plus motesanib 125 mg QD. DLTs occurred only in cohort 6 (one patient had grade 3 fatigue and one had grade 3 rash). The MTD for motesanib in combination with erlotinib only was established as 125 mg QD. Enrollment in cohort 2 was suspended as a result of the increased risk of cholecystitis observed at the 75-mg BID dose level in other motesanib studies [16,20].

### Adverse events

Of the 48 patients who received motesanib, 40 (83%) experienced motesanib-related adverse events, most commonly diarrhea, nausea, vomiting, fatigue, and anorexia (Table 2). Several adverse events of specific interest considered related to motesanib treatment occurred and included grade ≤ 3 hypertension, grade 3 and 4 neutropenia, grade 3 deep vein thrombosis, grade 4 pulmonary embolism, and grade 3 cholecystitis (Table 2). Twenty-three patients experienced grade ≥ 3 adverse events related to motesanib treatment, primarily in cohort 3 (eight of 10 patients; 125 mg QD motesanib plus gemcitabine and erlotinib) and in cohort 6 (four of seven patients; 125 mg QD motesanib plus erlotinib). No grade 5 motesanib-related adverse events occurred during the study. Fourteen patients (29%) had serious motesanib-related adverse events, which included nausea (n = 5), vomiting (n = 5), deep vein thrombosis (n = 2), diarrhea (n = 2), pulmonary embolism (n = 2), and tumor necrosis (n = 2). Of those, seven patients were enrolled in cohort 3. Adverse events with a worst grade of 3 or higher considered related to gemcitabine or erlotinib occurring in the control cohort were anemia (grade 3; n = 1), febrile neutropenia (grade 3; n = 1), fatigue (grade 3; n = 1), and rash (grade 3; n = 1). There were no incidences of hypertension or thromboembolic events in the control cohort.

**Table 2:** Patient incidence of motesanib-related adverse events

	Motesanib + Gemcitabine + Erlotinib (100 mg QD)				Motesanib + Erlotinib (150 mg QD)	
		
Patient Incidence	Motesanib 50 mg QD (n = 7)	Motesanib 100 mg QD (n = 8)	Motesanib 125 mg QD (n = 10)	Motesanib 75 mg BID (n = 9)	Motesanib 100 mg QD (n = 7)	Motesanib 125 mg QD (n = 7)
Any adverse event, n	3	8	9	7	7	6
Adverse events of grade 3, n**^a^**	0	3	6	3	3	4
Diarrhea	0	1	1	0	2	1
Nausea	0	0	3	1	0	0
Vomiting	0	1	2	0	0	0
Fatigue	0	1	0	0	0	2
Tumor necrosis	0	0	0	1	1	0
Deep vein						
thrombosis	0	0	2	0	0	0
Abnormal liver						
function test	0	1	0	0	0	1
Neutropenia	0	1	0	0	1	0
Adverse events of grade 4, n						
Pulmonary	1	0	2	1	0	0
embolism	0	0	2	0	0	0
Febrile neutropenia	1	0	0	0	0	0
Neutropenia	0	0	0	1	0	0
Adverse events of interest and highest (worst) grade, n						
Hypertension	0	0	0	2	1	2
Grade 3	0	0	0	0	0	1
Thrombophlebitis (all grade 3)	0	0	1	0	0	0
Gallbladder toxicity	0	0	2^b^	0	1^c^	0
Grade 3	0	0	1	0	0	0
Hemorrhagic events	0	0	1^d^	0	1^e^	0
Grade 3	0	0	1	0	0	0
Cardiac toxicity (all grade 3)	0	1^f^	0	0	0	0

BID = twice daily; QD = once daily

^a^For motesanib-related adverse events only those grade 3 events are listed that occurred in ≥ 2 patients.

^b^Cholecystitis (n = 1; grade 3) and gallbladder enlargement (n = 1; grade 1)

^c^Gallbladder enlargement (grade 2)

^d^Subdural hematoma

^e^Epistaxis (grade 1)

^f^Congestive heart failure and pulmonary edema

Of the 56 patients who received erlotinib, 54 (96%) experienced erlotinib-related adverse events, most frequently rash (n = 42), diarrhea (n = 35), nausea (n = 23), anorexia (n = 16), vomiting (n = 15), and fatigue (n = 14). Among patients in cohorts 5 and 6 (motesanib plus erlotinib without gemcitabine), the most common adverse events were diarrhea (n = 10), rash (n = 9), nausea (n = 7), fatigue (n = 5), anorexia (n = 4), and vomiting (n = 4).

Adverse events leading to study discontinuation included pulmonary embolism (n = 3) and fatigue (n = 2). One patient discontinued the study because of serious grade 3 cholecystitis. No patients withdrew from the study because of thrombotic events. Eight deaths occurred during the study, none of which were considered related to any study drug treatment. Six were attributed to disease progression; the causes for the other two deaths were pneumonia and sepsis. Overall, 24 patients (50%) had at least one interruption in motesanib treatment and 14 (29%) had at least one dose reduction as a result of adverse events. Among patients who received motesanib plus gemcitabine and erlotinib, 35% had at least one dose interruption and 26% had at least one dose reduction due to adverse events (36% and 36%, respectively, among patients who received motesanib plus erlotinib).

### Pharmacokinetics

**Figure 2:**
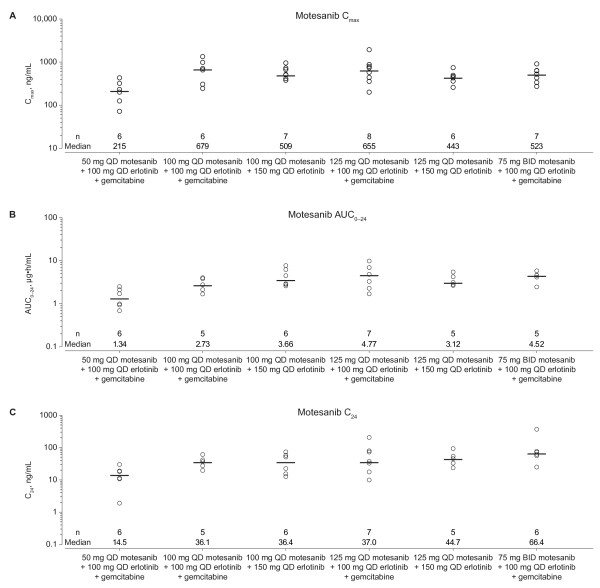
**Motesanib pharmacokinetic (PK) parameter estimates across cohorts**. Individual (circles) and median (lines) values are shown by motesanib dose cohort for C_max _(**A**), AUC_0—24 _(**B**), and C_24 _(**C**) values after oral administration of motesanib in combination with 100 mg QD erlotinib and gemcitabine or 150 mg QD erlotinib alone on week 3. Data for all subjects who received the protocol-specified treatment are shown.

Pharmacokinetic parameter estimates of motesanib in combination with 100 mg QD erlotinib and gemcitabine or in combination with 150 mg QD erlotinib alone are shown in Figure 2 according to motesanib dose cohort. After QD or BID administration in combination with 100 mg erlotinib and gemcitabine at week 3, motesanib was rapidly absorbed. Overall median t_max _values ranged from 0.6 to 2 hours. The mean estimated terminal elimination half-life (t_1/2, z_) ranged from 4.8 to 8.6 hours. At week 3, the C_max_, AUC_0—24_, and C_max _at 24 hours postdose (C_24_) in the dose range of 50 to 125 mg QD were generally within the range of values observed in a study of motesanib monotherapy [16]. Similarly, estimates of C_max_, AUC_0—24_, and C_24 _at the 75-mg BID dose were within the range observed at this dose in a study of motesanib in combination with gemcitabine [18].

After QD administration in combination with 150 mg erlotinib at week 3, motesanib was rapidly absorbed. The overall median t_max _value was 2 hours, and the mean estimated t_1/2, z _for motesanib ranged from 6.0 to 7.7 hours. The C_max _values at week 3 for the 100 mg QD dose were within the range of values observed in a study of motesanib monotherapy [16], but the AUC_0—24_, and C_24 _values were slightly (< 2-fold) higher, although high intersubject variability was observed in this study (Figure 2). However, the C_max_, AUC_0—24_, and C_24 _values at week 3 for the 125-mg QD dose were within the range observed at this dose in a monotherapy study [16].

Following QD administration of 100 mg erlotinib in combination with gemcitabine, erlotinib had a median t_max _ranging from 2 to 6 hours at week 2. Mean t_1/2, z _ranged from 17 to 33 hours. After QD administration of erlotinib in combination with gemcitabine and motesanib at week 3, erlotinib had an overall median t_max _value of 2 hours. Mean t_1/2, z _values ranged from 12 to 19 hours. Based on the GLSM estimates for the ratio of week 3 to week 2 erlotinib parameter values, erlotinib exposure was approximately 20% to 35% as assessed by C_max _and 10% to 50% as assessed by AUC_0—24 _(Table 3). The reduction in erlotinib exposure did not appear to be dependent on motesanib dose.

**Table 3:** Pharmacokinetics of erlotinib after single-dose administration in combination with motesanib

		AUC_0—24_		C_max_
Dose Cohort	n	GLSM Ratio Week 3: Week 2^a^ (90% CI)	n	GLSM Ratio Week 3: Week 2^a^ (90% CI)
50 mg motesanib QD + 100 mg erlotinib QD + gemcitabine	7	0.49 (0.25-0.96)	7	0.65 (0.39-1.08)
100 mg motesanib QD + 100 mg erlotinib QD + gemcitabine	7	0.53 (0.25-1.11)	8	0.82 (0.49-1.37)
125 mg motesanib QD + 100 mg erlotinib QD + gemcitabine	9	0.91 (0.48-1.73)	10	0.73 (0.48-1.09)
75 mg motesanib BID + 100 mg erlotinib QD + gemcitabine	9	0.52 (0.37-0.74)	9	0.75 (0.54-1.05)
100 mg motesanib QD + 150 mg erlotinib QD	7	0.46 (0.34-0.60)	7	0.59 (0.41-0.84)
125 mg motesanib QD + 150 mg erlotinib QD	7	0.46 (0.19-1.13)	7	0.61 (0.50-0.73)

AUC_0—24 _= area under the curve from time 0-24 h; BID = twice daily; C_max _= peak plasma concentration; CI = confidence interval; GLSM = geometric least squares means; QD = once daily

^a^Week 3, erlotinib plus motesanib treatment; week 2, erlotinib treatment only.

Following QD administration of 150 mg erlotinib at week 2, erlotinib had a median t_max _ranging from 1 to 3 hours. Mean t_1/2, z _ranged from 25 to 33 hours. After QD administration of erlotinib and motesanib at week 3, erlotinib had median t_max _values from 2 to 4 hours and a mean t_1/2, z _value of approximately 8 hours. Based on the GLSM estimates for the ratio of week 3 to week 2 erlotinib parameter values, erlotinib exposure was reduced by approximately 40% lower for C_max _and approximately 50% lower for AUC_0—24 _(Table 3), but did not appear to be dependent on motesanib dose.

Motesanib trough concentrations were similar across patients at each dose level tested, regardless of treatment. Erlotinib trough concentrations appeared to decrease in the presence of motesanib compared with erlotinib alone.

### Tumor response

**Figure 3:**
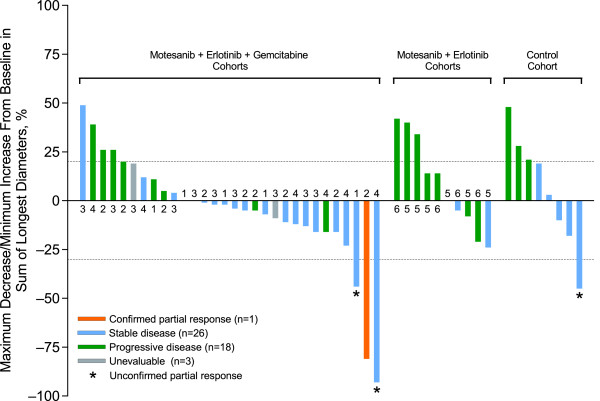
**Maximum change from baseline in the sum of the longest diameters of target lesions (as assessed by the investigator per RECIST)**. "Unevaluable" refers to patients who had a response assessment before the scheduled first assessment without an additional postbaseline assessment. 1 = 50 mg QD + gemcitabine/erlotinib; 2 = 75 mg BID + gemcitabine/erlotinib; 3 = 125 mg QD + gemcitabine/erlotinib; 4 = 100 mg QD + gemcitabine/erlotinib; 5 = 100 mg QD + erlotinib; 6 = 125 mg QD + erlotinib.

Of 49 patients with measurable disease (per RECIST) at baseline, one patient with NSCLC (75 mg motesanib BID plus gemcitabine and erlotinib) achieved a confirmed partial response. Three patients with the following tumor types had unconfirmed partial responses: pancreatic cancer (control), NSCLC (50 mg QD motesanib plus gemcitabine and erlotinib), and anaplastic thyroid cancer (100 mg QD motesanib plus gemcitabine and erlotinib). The latter patient had a 93% reduction from baseline in tumor dimensions at the first tumor assessment (week 12) but was subsequently assessed as having progressive disease (week 24) based on an increase in tumor dimensions of > 20% per RECIST. At that time, tumor dimensions were still reduced by 89% from baseline. Twenty-six patients, primarily in the cohorts that received the triple combination as well as in the control, had stable disease as a best tumor response; of those, three had durable stable disease with a duration of ≥ 24 weeks from study day 1 (125 mg QD and 75 mg BID motesanib plus gemcitabine and erlotinib, 125 mg QD motesanib plus erlotinib; n = 1 each). The most common tumor types in patients who achieved stable disease were colon cancer (n = 4) and NSCLC (n = 2). Maximum changes from baseline in tumor measurements are shown in Figure 3, indicating that most responses of stable disease were associated with modest tumor regression. A total of 25 patients (44%) had reductions in tumor size from baseline.

## Discussion

The primary objectives of the current study were to determine the target dose or MTD and characterize the safety of motesanib administered QD or BID in combination with erlotinib and gemcitabine in patients with advanced solid tumors. In previous studies, 125 mg QD was the MTD of motesanib administered as monotherapy [16] and was the target dose in combination with gemcitabine [18]. In the current study, the MTD for motesanib plus erlotinib and gemcitabine was established as 100 mg QD. At a dose of 125 mg QD, motesanib was not tolerable in the triple combination and was associated with a higher incidence of DLTs and serious adverse events, including thromboembolic events (pulmonary embolism and deep vein thrombosis), which were not observed in other dosing cohorts. However, at the 125-mg QD dose motesanib was tolerable when combined with erlotinib (150 mg QD) only. The motesanib 75-mg BID dosing cohort was suspended when an increased risk of cholecystitis was found at that dose level in other studies of motesanib as a monotherapy [16,17] or in combination with carboplatin/paclitaxel and an EGFR inhibitor [20]. Cholecystitis is not considered a common class effect of VEGF(R) inhibitors, although three other small-molecule inhibitors (sunitinib, cediranib, and sorafenib) have reported its occurrence [2,22,23]. No gallbladder toxicities were observed in the BID cohort of the present study, but one patient (with malignant mesothelioma) in the motesanib 125-mg QD cohort experienced serious grade 3 cholecystitis. The patient presented with abdominal pain approximately 26 days after initiation of motesanib treatment and was diagnosed with acute acalculous cholecystitis. A laparoscopic cholecystectomy was performed, which resulted in complete resolution of the symptoms, and the patient withdrew from the study. The cholecystitis was considered to be related to motesanib treatment and not related to erlotinib or gemcitabine treatment.

Overall, the incidence and severity of the most frequently occurring motesanib-related adverse events (diarrhea, nausea, vomiting, fatigue, and anorexia) were consistent with those observed in other studies of motesanib as monotherapy [16,17], in combination with chemotherapy [18], and in combination with an EGFR inhibitor and chemotherapy [20,24]. Skin toxicities are frequently associated with the use of many EGFR inhibitors [25]. In the present study, the incidence of erlotinib-related skin rash was 75%, which is similar to the 72% incidence rate that was reported in a phase 3 combination study of erlotinib and gemcitabine for the treatment of metastatic pancreatic cancer [13]. There did not appear to be any exacerbation of erlotinib-related skin toxicity with motesanib coadministration. A number of motesanib-related adverse events of interest occurred, including hypertension, thromboembolic events, cholecystitis, and neutropenia. Most of these events are considered class effects [26] and have been described previously with motesanib treatment [16-20,24]. In the current study we observed an increased incidence and severity of these adverse events in the 125-mg QD cohort of the triple combination arm.

The pharmacokinetics of motesanib were not markedly affected by the combination with erlotinib and gemcitabine, or with erlotinib only. However, erlotinib C_max _and AUC_0—24 _appeared to be lower following either combination treatment (motesanib plus erlotinib and gemcitabine or motesanib plus erlotinib alone). Pharmacokinetic interactions between motesanib and erlotinib may have occurred because motesanib is an inhibitor of cytochrome P450 (CYP) 3A4 and an inducer of CYP1A2 [27]. Erlotinib is metabolized at least in part by CYP3A4 and CYP1A2 [28]. Hence, the observed decrease in erlotinib C_max _and AUC_0—24 _after coadministration with motesanib may have resulted from induction of CYP1A2 by motesanib. It has previously been reported that coadministration of gefitinib and sorafenib results in reduced exposure to gefitinib but not sorafenib [29]. Pharmacokinetic interactions with gemcitabine were not expected because it is primarily metabolized by deoxycytidine deaminase [30]. Taken together the data show that although there are no pharmacokinetic interactions between gemcitabine and either motesanib or erlotinib, interactions occur when motesanib and erlotinib are coadministered. Dose modifications of erlotinib may require further investigation when given in combination with motesanib.

In the present study, tumor response was an exploratory endpoint. One confirmed and three unconfirmed partial responses were observed, all of which but one unconfirmed response occurred in the triple combination arm. Most patients (70%) receiving motesanib 125 mg QD plus gemcitabine and erlotinib achieved stable disease, but this cohort also experienced more toxicities. Furthermore, reductions in tumor size associated with stable disease were largely modest across treatment cohorts. The risk/benefit ratio of treatment with a VEGF pathway inhibitor plus an EGFR inhibitor and chemotherapy has recently been highlighted in an ongoing phase 2 study in NSCLC, in which 48% of patients achieved a partial response and 22% achieved stable disease. However, 36% of patients had grade 4 adverse events [31]. Recent studies have suggested that the combination of a VEGF pathway inhibitor and an EGFR inhibitor may provide clinical benefit in some settings, but the results have not been uniformly positive and further investigation is, therefore, warranted. In a phase 3 study, second-line treatment with bevacizumab plus erlotinib in NSCLC did not extend overall survival (the primary endpoint) compared with erlotinib plus placebo although progression-free survival and objective response rate were improved [12]. In another phase 3 study in patients with NSCLC, treatment with bevacizumab plus erlotinib as maintenance therapy improved progression-free survival compared with bevacizumab among patients who received bevacizumab plus chemotherapy as first-line treatment [32]. A phase 3 study of bevacizumab plus erlotinib and gemcitabine in patients with metastatic pancreatic adenocarcinoma did not show an increase in overall survival, compared with control (erlotinib, gemcitabine, and placebo), but reported a significant increase in progression-free survival [33].

## Conclusions

In conclusion, in patients with solid tumors, motesanib in combination with gemcitabine and erlotinib was tolerable at the MTD of 100 mg QD. Motesanib 125 mg QD was tolerable in combination with erlotinib only. The pharmacokinetics of motesanib were not markedly affected by combination treatment with erlotinib and gemcitabine; however, erlotinib exposure was reduced when coadministered with motesanib. Tumor responses were observed but additional studies are required to evaluate whether the triple combination of motesanib plus gemcitabine and erlotinib or the double combination of motesanib plus erlotinib provides clinical benefit in specific tumor types.

## Abbreviations

λ_Z_: Terminal elimination rate constant; AUC: area under the concentration versus time curve; AUC_0—24_: area under the concentration versus time curve from time 0 to 24 hours; BID: twice daily; C_24_: maximum observed plasma concentration at 24 hours postdose; C_max_: maximum observed plasma concentration; CI: confidence interval; CTCAE: Common Terminology Criteria for Adverse Events; CYP: cytochrome P450; DLT: dose-limiting toxicity; EGFR: epidermal growth factor receptor; GLSM: geometric least squares means; HR: hazard ratio; LC/MS/MS: liquid chromatography/tandem mass spectometry; LLOQ: lower limit of quantitation; MTD: maximum tolerated dose; NSCLC: non—small cell lung cancer; QD: once daily; RECIST: Response Evaluation Criteria in Solid Tumors; t_1/2, z_: estimated terminal elimination half-life; t_max_: time to reach the maximum observed plasma concentration; VEGF: vascular endothelial growth factor; VEGFR: vascular endothelial growth factor receptor.

## Competing interests

DK has received commercial research grants and support from Amgen Inc. NT and TP have served as consultants/advisors for Amgen Inc. JD has as served on the speakers' bureau for Novartis and Pfizer; has served as a consultant/advisor for Novartis, Pfizer, and Merck; and has provided testimony for Pfizer. SW has served on the speakers' bureau for Roche and Schering-Plough and has served as a consultant/advisor for Roche. SM, YNS, JJ, AHA are employees and shareholders in Amgen, Inc. LLS declares that she has no competing interests.

## Authors' contributions

DK contributed to study concept and design, data acquisition, and data analysis; critically reviewed the manuscript and gave final approval of the version to be published. NT contributed to data acquisition, data analysis and interpretation; critically reviewed the manuscript and gave final approval of the version to be published. JD contributed to data acquisition and data interpretation; revised the manuscript critically for important intellectual content and gave final approval of the version to be published. SW contributed to data acquisition; critically reviewed the manuscript and approved the final version to be published. LLS contributed to data acquisition; critically revised the manuscript and approved the final version to be published. SM performed the statistical data analysis, critically reviewed the manuscript and approved the final version to be published. YNS contributed to the study concept and design, performed the pharmacokinetic analysis, critically revised the pharmacokinetic section of the manuscript and approved the final version to be published. JJ contributed to the pharmacokinetic analysis and approved the final manuscript version to be published.

AHA contributed to the study concept and design and the data analysis; critically revised the manuscript and approved the final version to be published.

TP contributed to study concept and design, data acquisition, data analysis and the writing of the manuscript; and approved the final version to be published.

## Pre-publication history

The pre-publication history for this paper can be accessed here:

http://www.biomedcentral.com/1471-2407/11/313/prepub
